# INVESTIGATING SOCIAL DETERMINANTS OF HEALTH IN ASIAN AMERICANS AND PACIFIC ISLANDERS

**DOI:** 10.1093/geroni/igae098.0375

**Published:** 2024-12-31

**Authors:** Yaguang Zheng, Yanping Jiang, Bei Wu

**Affiliations:** Chair; Co-Chair; Discussant

## Abstract

Asian Americans and Pacific Islanders (AAPI) are one of the fastest-growing racial minority groups in the United States, who disproportionately experience some unfavorable social determinants of health (SDoH), such as limited English proficiency, unemployment, lack of health insurance, low health literacy, lack of culturally and linguistically sensitive healthcare providers and programs, and reduced healthcare access. However, limited research has explored the impacts of SDoH on health outcomes among this population. The objective of this symposium is to address knowledge gaps by examining the association of SDoH with various health outcomes in AAPI. Jiang and colleagues explore the immigration stressors and sleep health among older Chinese immigrants using the longitudinal data from the Population Study of Chinese Elderly in Chicago. Qi and colleagues examine the prospective associations of multiple SDoH with premature all-cause mortality and investigate their respective contributions among a nationally representative cohort of Asian Americans. Further, Wang et al. discuss the challenges and successes in recruiting Chinese and Korean American dementia caregivers living in minority and underrepresented communities for a large health intervention study. Lastly, Kim & colleagues examine the end-of-life care disparities for AAPI Medicare beneficiaries with dementia using Medicare data. Following the paper presentations, Dr. Wu will lead a discussion. The novel findings from this symposium will provide a comprehensive understanding of how SDoH impacts health outcomes in AAPI and how unique SDoH can be particularly targeted to improve health equity for this underserved ethnic minority group.

